# Forsaken Foregut: Case Report of Simultaneous Black Esophagus and Ischemic Cholangiopathy

**DOI:** 10.1155/2017/8362613

**Published:** 2017-04-24

**Authors:** Paul A. Cameron, Franzjosef Schweiger

**Affiliations:** ^1^Dalhousie University, Queen Elizabeth II Health Sciences Centre, Halifax, NS, Canada B3H 2Y9; ^2^Dalhousie University, 1276 South Park Street, Rm 483 Bethune Building, Halifax, NS, Canada B3H 2Y9

## Abstract

Black esophagus or acute esophageal necrosis rarely occurs after severe hemodynamic compromise or low-flow states. Other contributing factors may include corrosive injury from gastric contents and diminished mucosal repair mechanisms. Ischemic cholangitis, another rare clinical entity, is also usually the result of a significant vascular and/or hypotensive insult to the biliary tree. We describe the first case of combined acute esophageal necrosis and ischemic cholangiopathy in a 62-year-old male who completely recovered from the esophageal injury but developed progressive liver failure from ischemic cholangiopathy.

## 1. Case

A 62-year-old man with a history of coronary artery disease, hypertension, dyslipidemia, type II diabetes mellitus, and active alcohol use presented to the hospital after he was found unresponsive at home. A liver biopsy 8 years earlier for hepatitis C infection had shown stage 2 fibrosis at that time.

On admission he was hypovolemic and hypothermic and had an acute kidney injury. He was confused and jaundiced. He had no stigmata of chronic liver disease. His abdomen was distended and he lacked pedal pulses bilaterally. A nasogastric tube was inserted and coffee ground-like fluid was aspirated confirming presence of upper gastrointestinal bleeding. Initial investigations revealed mild leukocytosis (17.4 × 10^9^/L) with 64% bands, elevated INR (2.0), and creatinine (302 umol/L). Totally bilirubin was 190 mmol/L and ALT was 101 U/L and alkaline phosphatase was 136 U/L. Serum hepatitis C RNA was negative.

**Figure 1:**
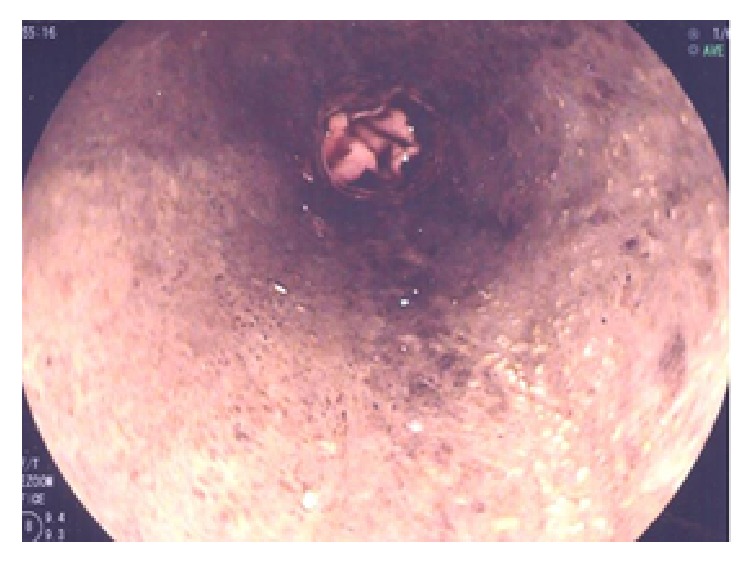
Black esophagus on endoscopy.

Upper GI endoscopy demonstrated black mucosa from the proximal esophagus to the gastroesophageal junction. There were no varices, ulcerations, or other causes of bleeding (Figure 1). Biopsies showed acute necrotizing esophagitis with diffuse brown pigment.

He improved clinically with no further bleeding and he attained normal renal function within a week. Repeat upper GI endoscopy five weeks later demonstrated a completely normal esophagus with no stricture. His subsequent hospital course was complicated by ischemic ulcerations and osteomyelitis of the right lower extremity eventually requiring amputation despite antibiotic treatment.

**Figure 2:**
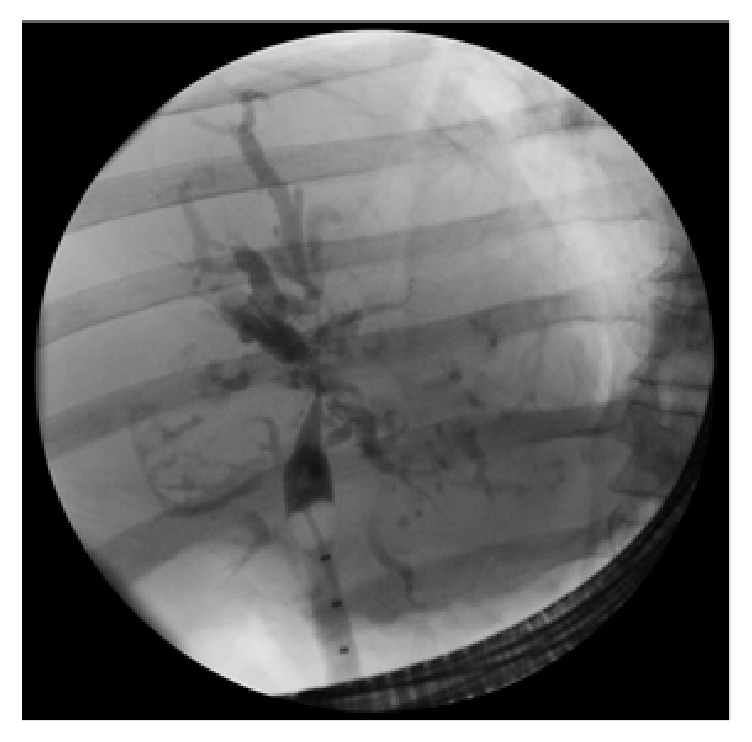
Endoscopic Retrograde Cholangiopancreaticogram showing sclerosing cholangitis.

**Figure 3:**
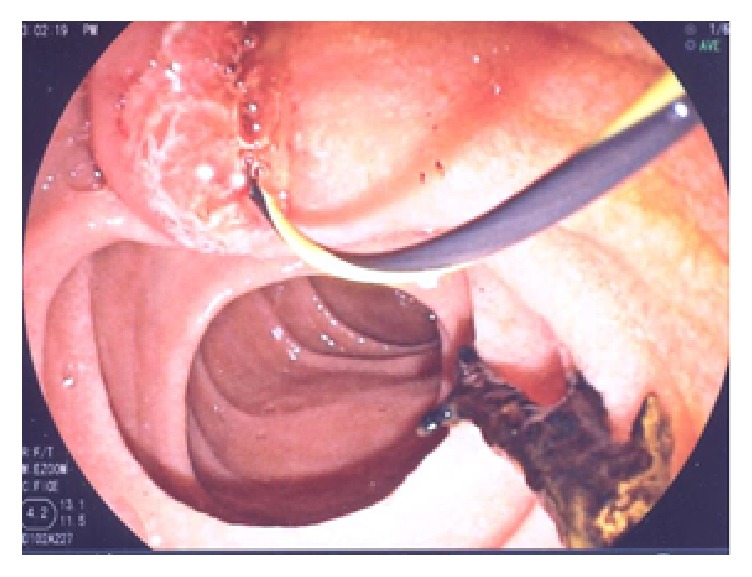
Endoscopic visualization of necrotic biliary cast.

Three months after initial presentation his bilirubin (total 69 mmol/L, direct 50 mmol/L), ALP (675 U/L), and gamma GT (508 U/L) were persistently elevated. Serial measurements of these markers are shown in Table 1. He experienced recurrent Gram-negative bacteremia; therefore, a CT of his abdomen was performed and revealed mildly dilated intrahepatic biliary ducts. ERCP showed cholelithiasis and choledocholithiasis and balloon occlusion cholangiogram suggested sclerosing cholangitis (Figure 2). One week following endoscopic sphincterotomy and clearance of choledocholithiasis repeat ERCP confirmed secondary sclerosing cholangitis and several biliary casts could be extracted by balloon sweeping of the biliary tree (Figure 3). Eight months after presentation he developed large volume ascites treated with diuretics.

## 2. Discussion

Acute esophageal necrosis or black esophagus (Gurvits Syndrome) is a disease characterized by diffuse, circumferential black appearing mucosa that always affects the distal mucosa due to relative hypovascularity [1, 2] Estimated incidence is between 0.008 and 0.28% of patients undergoing upper endoscopy based on autopsy and retrospective reviews [1, 3–5]. It occurs more frequently in older males with medical comorbidities such as diabetes, malignancy, hypertension, and alcohol abuse [3, 4, 6].

The etiology usually due to significant hemodynamic compromise or low-flow states resulting in esophageal hypoperfusion combined with corrosive injury from gastric contents and impaired mucosal repair mechanisms [4, 7, 8]. A large meta-analysis by Gurvits et al. found ischemia to be the most commonly implicated etiology [3]. The typical presentation consists of upper gastrointestinal bleeding, vomiting, and abdominal pain [4, 8, 9]. Persistent chest pain may reflect impending perforation that occurs in less than 7% of cases [3].

Our patient had several predisposing factors including older age, diabetes mellitus, alcoholism, and peripheral and coronary atherosclerotic disease associated with hemodynamic instability as the precipitating event. After resuscitation and a period of convalescence he was able to swallow without any impairment and complete healing of his esophageal mucosa was confirmed endoscopically. Case reports and retrospective analyses suggest the most common treatment modalities including proton pump inhibitors, sucralfate, histamine receptor antagonists, withholding of oral feeding, parenteral nutrition, and treatment of underlying infectious etiologies when present [3]. It is often reversible with supportive management [10].

Although he had a preexisting mild liver disease secondary to hepatitis C infection and possibly alcohol-related liver disease he developed a rapid injury to liver function over several months. This progressive deterioration occurred despite clearance of his bile duct of stones. The finding of biliary casts during ERCP is highly suggestive of ischemic cholangiopathy [11]. This clinical entity is one of several causes of secondary sclerosing cholangitis [12]. It is characterized by progressive increase in cholestatic biomarkers or recurrent bacterial cholangitis with a propensity to develop into liver failure over the course of several months [13–16]. Well known in the field of liver transplantation [17], ischemic cholangitis can be caused by any interference of blood flow to the peribiliary plexus including vascular injury during surgery, chemoembolization, low-flow states, or hypercoagulable conditions [11, 18, 19]. Recently it has been described in critically ill intensive care unit patients on high-pressure mechanical ventilation and vasopressor therapy [14].

The diagnosis is usually made by cholangiography showing multiple diffuse strictures of the biliary tree and intraductal filling defects representing biliary casts [15, 20]. The latter consist mainly of protein likely related to necrotic biliary epithelial cells and increase the risk of secondary bacterial cholangitis [19].

Our patient exhibited a typical presentation of both black esophagus and ischemic cholangiopathy likely resulting from severe low-flow state and shock in the context of underlying vasculopathy. Upon improvement of hemodynamic indices the recovery of esophageal function is characteristic of acute esophageal necrosis. However, the irreversibility and progression to decompensated cirrhosis in our patient are also typical of ischemic cholangiopathy and confer a poor prognosis [11].

## 3. Conclusion

While either of these diseases is rare their coexistence ought to be considered in patients with shock, signs of gastrointestinal hemorrhage, and elevated cholestatic liver biomarkers that fail to improve with amelioration of hemodynamic parameters.

## Figures and Tables

**Table 1:** Cholestatic biomarkers.

	ALP (U/L)	GGT (U/L)
Admission	136	140
1 week	230	259
2 weeks	379	226
4 weeks	464	154
2 months	675	
3 months	967	
4 months	780	508
5 months	724	1455
6 months	649	
7 months	638	
